# Discordance between measures of *Mycobacterium tuberculosis* sensitization and type 2 diabetes mellitus in the United States (NHANES): A population-based cohort study

**DOI:** 10.1016/j.jinf.2025.106496

**Published:** 2025-04-30

**Authors:** Itai M. Magodoro, Katalin A. Wilkinson, Brian L. Claggett, Ntobeko A.B. Ntusi, Mark J. Siedner M, Robert J. Wilkinson

**Affiliations:** a Department of Medicine, University of Cape Town, Observatory 7925, Republic of South Africa; b Centre for Infectious Diseases Research in Africa, University of Cape Town, Observatory 7925, Republic of South Africa; c Institute of Infectious Disease and Molecular Medicine, University of Cape Town, Observatory 7925, Republic of South Africa; d Francis Crick Institute, Midland Road, London NW1 1AT, United Kingdom; e Harvard Medical School, Boston 02115, MA, USA; f Cardiovascular Division, Brigham and Women’s Hospital, Boston 02115, MA, USA; g South African Medical Research Council, Tygerberg 7505, Republic of South Africa; h ARUA/GUILD Cluster of Research Excellence on Noncommunicable Diseases and Associated Multimorbidity; i Medical Practice Evaluation Center and Division of Infectious Diseases, Massachusetts General Hospital, Boston 02114, MA, USA; j Africa Health Research Institute, Mtubatuba 3935, Republic of South Africa; k University of KwaZulu-Natal, Durban 4013, South Africa; l Department of Infectious Diseases, Imperial College, London W12 0NN, United Kingdom

**Keywords:** LTBI, Tuberculin skin testing, IFN-γ Release, Diabetes

## Abstract

**Objective:**

We examined how latent TB infection (LTBI), evaluated by cell-mediated immune responses to *Mycobacterium tuberculosis* (*Mtb*) antigens, impacts glucose metabolism in US adults.

**Methods:**

*Mtb* sensitization was evaluated by interferon-γ (IFN-γ) release assay (IGRA+: assay reactivity) and tuberculin skin testing (TST+: skin induration ≥10 mm), and categorized as: IGRA-/TST- (TB uninfected controls); IGRA-/TST+; IGRA+/TST-; or IGRA+/TST+. Diabetes was ascertained by fasting plasma glucose (FPG) ≥7.0 mmol/L, HbA1c ≥6.5% and/or antidiabetic medication. Adjusted generalized additive models examined nonlinear effects of skin induration and IFN-γ reactivity on FPG and HbA1c; and LTBI on diabetes prevalence.

**Results:**

Among 1787 (IGRA-/TST-), 101 (IGRA-/TST+), 92 (IGRA+/TST-), and 99 (IGRA+/TST+) adults, skin induration linearly associated with FPG [effective degrees of freedom (EDF) =1.01; p < 0.001] and non-linearly with HbA1c [EDF=1.76; p=0.003]. IFN-γ reactivity correlated with neither FPG [p=0.58] nor HbA1c [p=0.94]. Relatedly, adjusted diabetes prevalence was greater in IGRA-/TST+ [24.9%; p=0.048] and IGRA+/ TST+ [27.3%; p=0.004] but not IGRA+/TST- [15.9%; p=0.69] individuals than among controls [15.3%].

**Conclusions:**

LTBI associated with glycemic measures and diabetes when assessed by skin induration, but not IFN-γ release. This suggests an association with innate immune activation rather than acquired T-cell response, as determined by *ex vivo* IFN-γ release assay.

## Introduction

Tuberculosis (TB) occurs as an immunological and pathological spectrum.^1,2^ The diversity of host immune responses to *Myco-bacterium tuberculosis* (*Mtb*) infection contributes to this heterogeneity, and to the resulting clinical forms and consequences of TB.^1^ Active TB, for example, has been linked to disordered energy metabolism, including type 2 diabetes mellitus (diabetes).^3,4,5,6^ Exaggerated and/or prolonged proinflammatory cytokine activation and deranged adipocyte, lipid and free fatty acid metabolism accompanying active TB are thought to alter cellular and intracellular insulin signaling in skeletal muscle, adipose tissue and the liver, causing insulin resistance (IR).^6^ Asymptomatic or latent *Mtb* infection (LTBI) lies at the other end of the tuberculosis spectrum. While there are 10 million new active TB cases annually, an estimated onequarter of the world’s population has LTBI.^7^ If and how LTBI impacts glucose metabolism is currently not well understood.

Unlike active disease, LTBI is not defined by direct detection of *Mtb*.^1^ Rather, it is inferred from the host’s immune reactivity to mycobacterial antigens. Current tests are based on quantifying the cell-mediated immune response using either skin induration from tuberculin skin testing (TST) or *ex vivo* interferon-γ (IFN-γ) reactivity to *Mtb*-specific antigens by IFN-γ release assays (IGRA).^8^ LTBI, encompassing individuals who are *Mtb*-sensitized, therefore may represent diverse immune responses to tuberculosis including eliminated, controlled or subclinical infection.^1,2^ How these immune states relate to glucose metabolism, including diabetes risk, has not been addressed. Because LTBI is a spectrum, knowledge of its innate and adaptive immune effectors may help elucidate the host factors underlying TB’s potential diabetogenicity.

In this study, we characterized associations between LTBI and glucose metabolism indices in adults. Specifically, we examined how measures of *Mtb* sensitization, skin induration in the TST and interferon-γ reactivity from IGRA, relate to fasting plasma glucose (FPG), glycated hemoglobin (HbA1c) and prevalent diabetes. Because these tests are based on different aspects of cell-mediated immunity, we hypothesized that comparing and contrasting their relationships with glycemia would shed light on the mechanisms potentially driving diabetes in LTBI.

## Methods

Our study was conducted and is reported in accordance with guidelines for Strengthening the Reporting of Observational Studies in Epidemiology (STROBE).^9^

### Study design and participants

We used publicly accessible data from the 2011–2012 NHANES cycle. The NHANES, fully described elsewhere,^10^ are biennial cross-sectional health surveys of non-institutionalized US adults. Participants are selected to be nationally representative through multistage probability cluster sampling and complete questionnaires, physical examination, and laboratory testing of their biological samples. Because NHANES data are de-identified, their analyses do not require prior institutional ethics review. For the present study, we included all adults aged ≥ 20 years old with complete data on fasting plasma glucose (FPG), glycated hemoglobin (HbA1c), IGRA, and TST.

### Procedures

#### Measures of Mtb sensitization and definition of latent tuberculosis infection (LTBI)

Participants underwent TST and IGRA testing. Tuberculin-purified protein derivative (PPD) product, Tubersol® (Sanofi, Bridgewater, NJ), was placed intradermally on the volar surface of the forearm and skin induration measured 48–72 h later. TST reading was standardized and blinded to participants’ medical history, including any prior contact with active TB cases. Venous blood was also drawn for IGRA using QuantiFERON-TB Gold In-Tube (QFT-GIT; Cellestis/Qiagen, Carnegie, Victoria, Australia). IGRA quantifies IFN-γ that is released from sensitized lymphocytes when whole blood is incubated with *Mtb* antigens (TB antigen tube) and compared to control (nil and mitogen) tubes.

LTBI was defined as skin induration (TST+) ≥10 mm^8^ or QFT-GIT (IGRA+) assay reactivity, that is: [nil] value IFN-γ ≤ 8.0 international units (IU)/mL, [TB antigen] minus [nil] value IFN-γ ≥0.35 IU/mL and [TB antigen] minus [nil] value IFN-γ ≥25% of the [nil] value.^11^ IFN-γ from the TB antigen-stimulated tubes (IFN-γ, hereafter) and skin induration were considered measures of *Mtb* sensitization, while the IFN-γ from unstimulated, negative control (nil) tubes (IFN-γ nil, hereafter) was considered a measure of general immune activation. Blood for IGRA was drawn prior to placement of the PPD. Both TST and IGRA results were used to define LTBI status as IGRA-/TST-; IGRA-/TST+; IGRA+/TST-; and IGRA+/TST+.

### Diabetes mellitus

Diabetes was defined according to standard criteria (2023) as any of FPG ≥ 7.0 mmol/L, HbA1c ≥ 6.5% or any self-reported use of oral hypoglycemic agents (OHA) and/or insulin.^12^ Data were not available to distinguish type 1 and 2 diabetes mellitus.

### Covariates

We extracted participants’ age, sex, race/ethnicity, socio-economic and health insurance coverage status, blood pressure (BP), body mass index (BMI), waist circumference, cigarette smoking and current use of prescribed medicines, including oral hypoglycemic agents (OHA) and insulin. Participants reported their race/ethnicity as any of Hispanic, non-Hispanic white, non-Hispanic black, and non-Hispanic Asian/Other. The household income-to-poverty ratio (PIR) was used to assess socio-economic status. Households with PIR ≤1.3 were considered to be living in poverty.^13^ Health insurance was current if a participant had coverage in the preceding 12 months. Participants were defined as current smokers if they had smoked at least 100 cigarettes in their lifetime and reported any smoking in the last 30 days. Non-smokers were those who either never smoked more than 100 cigarettes in their lifetime or had smoked ≥100 cigarettes in their lifetime but reported no smoking in the preceding 30 days.^14^ We defined hypertension as any current use of anti-hypertensive medicines and/or systolic blood pressure (SBP) ≥140 mmHg and/or diastolic blood pressure (DBP) ≥90 mmHg. HIV seroprevalence was 0.5% and was not factored in analyses.

### Statistical analysis

#### Primary analysis

We performed a complete case analysis based on our study’s inclusion criteria of nonmissing FPG, HbA1c, IGRA and TST. Our outcome of interest in the primary analysis was glucose metabolism, which we modeled as (dichotomous) prevalent diabetes and (continuous) FPG and HbA1c. Our exposures were (categorical) LTBI status and (continuous, i.e., size of skin induration from TST and IFN-γ quantity from IGRA) measures of *Mtb* exposure. Because our primary goal was not to estimate population-level parameters but to understand the relationships between glucose metabolism indices and measures of *Mtb* exposure, we did not incorporate inverse probability sampling weights into our analyses.

We summarized participants’ socio-demographic and clinical characteristics according to LTBI status, reporting mean (standard deviation, SD), median (interquartile range, IQR) or number (percent), as appropriate. The distributions of FPG and HbA1c (Kolmogorov-Smirnov test P ≥0.21) were separately plotted by LTBI status and their differences assessed by analysis of variance (ANOVA), repeated t-tests and linear regression adjusted for age, sex, race, health insurance coverage, household poverty income ratio (PIR), waist circumference and smoking status.

Next, generalized additive models (GAM) were applied to explore the relationships between FPG and HbA1c as outcomes and skin induration and IFN-γ as predictors. GAM, unlike traditional models, are flexible as they do not require specifying the form of the relationship (linear, quadratic, etc.) between the predictors and the response variable.^15^ Models included an interaction term between skin induration and IFN-γ and were formulated as below:

$$\begin{array}{l} \text{biomarker}\;={\text{s}}_{1}\cdot (\;\text{IFN}\;-\text{γ})+{\text{s}}_{2}\cdot (\text{TST}\;)+{\text{s}}_{3}\cdot (\;\text{IFN}\;-\text{γ},\;\text{TST}\;)+{\text{β}}_{1}\cdot \;\text{age}\;+{\text{β}}_{2}\cdot \;\text{sex}\;+\\ {\text{β}}_{3}\;•\text{race}\;+\ldots {\text{β}}_{4}\;•\text{smoking}\; \end{array}$$

GAM were also applied with diabetes (yes/no) as the response variable, and were similarly adjusted. These models reported predicted probabilities of having diabetes which we interpreted as expected or adjusted prevalences. Three-dimensional (3D) perspective plots were used to visualize the relationships between expected diabetes prevalence for given skin induration and IFN-γ. To ascertain the robustness of these models, we repeated the analysis modeling the association between diabetes and categorical LTBI status using generalized linear models (GLM) adjusted for the same covariates as the GAM. Diabetes prevalence and prevalence ratios for each LTBI category were derived from postestimation margins from these models.

### Secondary analysis

Because the relationship between TB and diabetes is probably bidirectional, we also explored how glucose metabolism might impact measures of *Mtb* sensitization among participants with LTBI, i.e., with either IGRA+ and/or TST+. This entailed estimating correlations between FPG and HbA1c, on one hand, and TST and IFN-γ, on the other, using Kendall’s Tau τ coefficients. The strength of correlation was determined to be either weak (0–0.39), moderate (0.40–0.69), or strong (0.70–1.0). Next, we modeled TST and IFN-γ as dependent on FPG and HbA1c using GAM adjusted for age, sex, race, health insurance coverage, household poverty income ratio (PIR), waist circumference and smoking status.

Analyses and visualizations were conducted using R, version 3.6.3 (R Foundation for Statistical Computing, Vienna, Austria), including the “*mgcv”* package for GAM^15^; Stata version 17.0 (StataCorp, College Station, TX, USA); and Inkscape’s vector graphics editor (Inkscape Project. (2020). All probability values were 2 sided with p-values < 0.05 considered indicative of statistical significance after Bonferroni correction, where indicated.

### Role of the funding source

The funders had no influence over analysis or publication of these results.

## Results

### Characteristics of study participants

A total of 2079 adults (≥20 years old) with complete FPG, HbA1c, IGRA and TST results constituted the analytic sample (Fig. E1). Of these, 1787 (86.0%) were IGRA-/TST- (i.e., TB uninfected controls), 101 (4.9%) were IGRA-/TST+, 92 (4.4%) were IGRA+/TST- and 99 (4.7%) were IGRA+/TST+. Their demographic and clinical characteristics, including skin induration and IFN-γ results, are summarized in Table 1. Mean (SD) skin induration according to LTBI status was 0.67 (1.9) mm for controls, 13.5 (3.0) mm for IGRA-/TST+, 2.7 (3.8) mm for IGRA+/TST-, and 15.5 (3.6) mm for IGRA+/TST+. The corresponding mean (SD) for IFN-γ was 0.08 (0.11) IU/mL, 0.15 (0.16) IU/mL, 2.7 (3.2) IU/mL and 5.1 (4.4) IU/mL, respectively. Immune activation as measured by IFN-γ nil was greater in those with *Mtb* sensitization (≥0.10 [0.14] IU/mL) compared to controls (0.06 [0.10] IU/mL; p=0.008).

Participants with LTBI, who were either IGRA+ and/or TST+, were generally older and more frequently of Hispanic race/ethnicity than those without TB infection (Table 1). They were less likely to hold health insurance. Rates of household poverty were comparable across the groups. While all four groups had comparable rates of hypertension and current smoking, as well as similar levels of obesity, participants who were IGRA+/TST+ and IGRA+/TST- had the highest systolic BP and highest rates of insulin and oral hypoglycemic medication use.

### Distribution of fasting plasma glucose and glycated hemoglobin

**Fig. 1:**
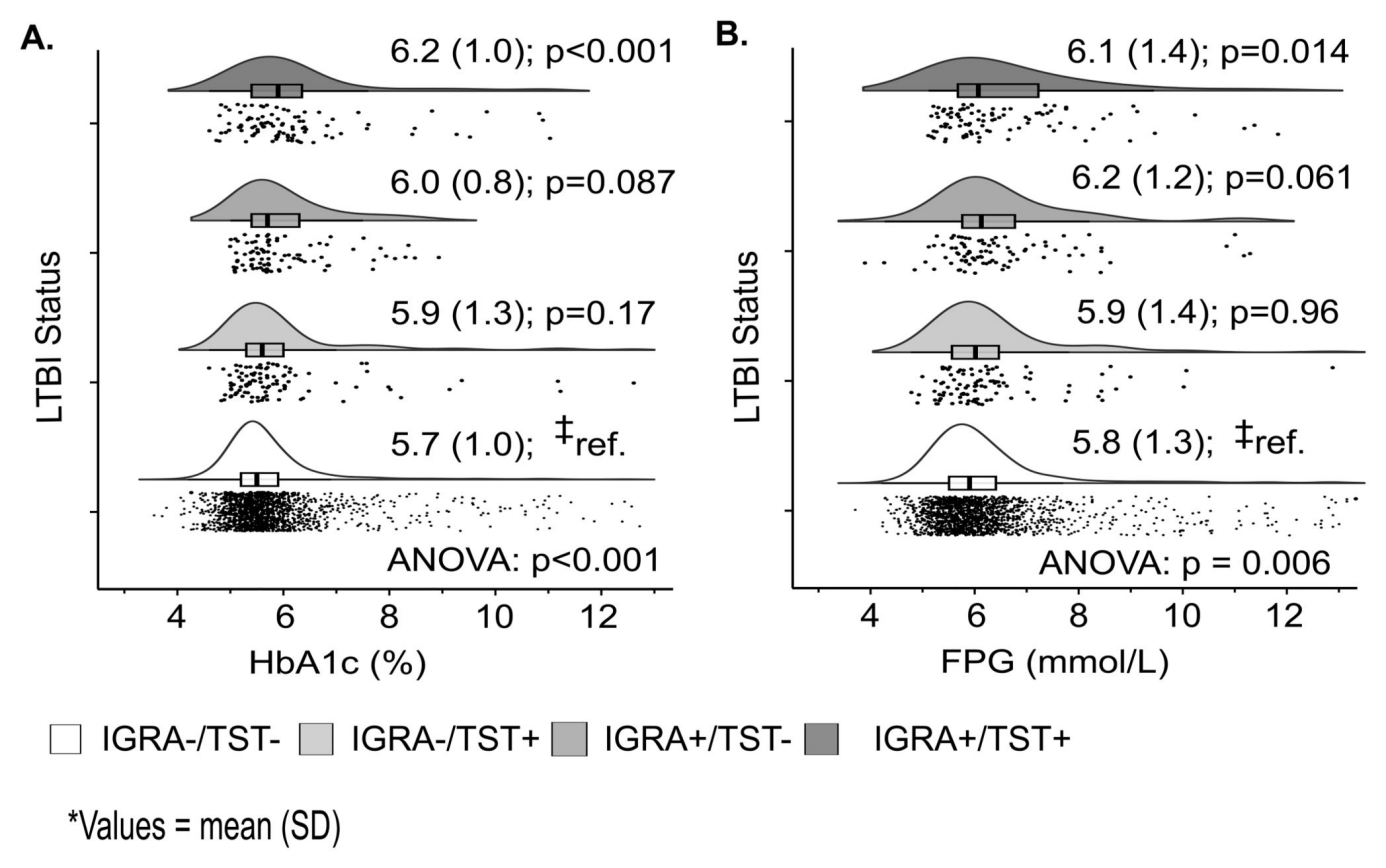
Biomarkers of glucose metabolism stratified by mycobacterial sensitization status, unweighted US NHANES 2011–2012 sample. ^‡^ Bonferroni adjusted p-values for repeated t-tests for mean differences in reference to the IGRA-/TST- group. HbA1c = glycated hemoglobin; FFG = fasting plasma glucose. Mycobacterial sensitization is defined by tuberculin skin testing (TST) and interferon-γ release assay (IGRA).

**Fig. 2:**
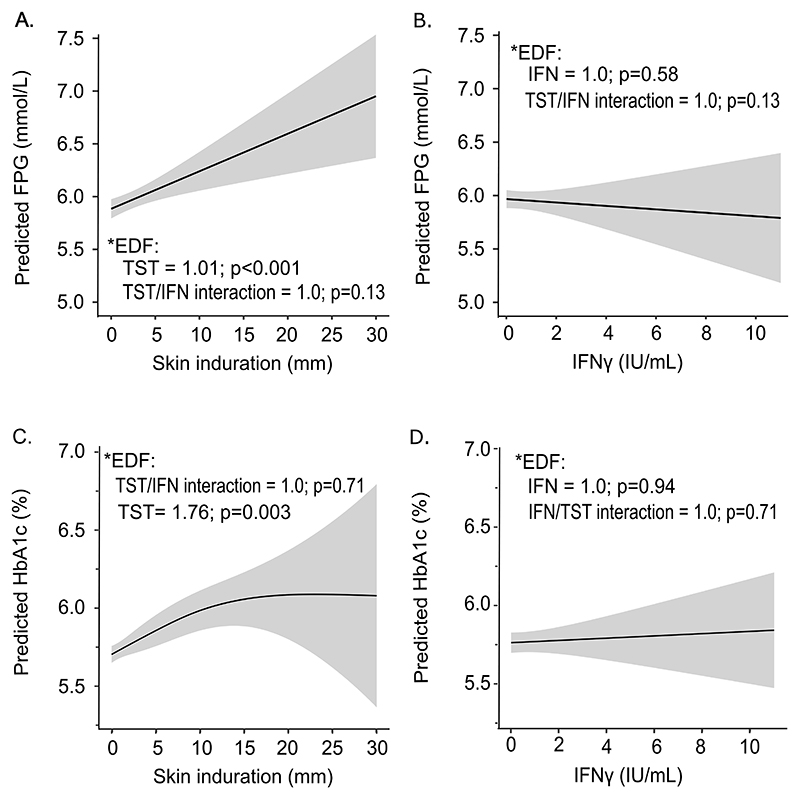
Predicted associations between fasting plasma glucose and HbA1c and measures of mycobacterial sensitization, unweighted US NHANES 2011–2012 sample. * EDF = effective degrees of freedom. Generalized additive models (GAM) of FPG and HbA1c on TST and IFN-γ, and including TST/IFN-γ interaction. *FPG (or HbA1c) =s_1_.(IFNγ) + s_2_.(TST) + s_3_. (IFNγ, TST) + β_1_.age + β_2_.sex + β_3_.race + … β_4_.smoking*. Gray areas represent 95% CI. Adjusted for age, sex, race, health insurance coverage, household poverty income ratio (PIR), waist circumference and smoking status.

FPG and HbA1c profiles of participants are summarized in Fig. 1A-B according to LTBI status. Adjusted and unadjusted general additive models of FPG and HbA1 in relation to skin induration and IFN-γ are shown, respectively, in Fig. 2A-D and Fig. E2 and, while adjusted mean differences (adjMD) in FPG and HbA1c between different LTBI types versus TB uninfected controls are presented in eTable 1. Mean HbA1c was higher in IGRA-/TST+ [adjMD (95%CI): 0.25 (0.05, 0.47)%; p=0.017] and IGRA+/TST+ [0.26 (0.04, 0.43)%; p=0.027] than controls (eTable 1). This was also the case for mean FPG, which was higher in IGRA-/TST+ [0.48 (0.12, 0.84) mmol/L; p=0.008] and IGRA+/TST+ [0.22 (0.0, 0.43) mmol/L; p=0.051] than in controls. Of note, participants who were IGRA+/TST- had comparable HbA1c [0.14 (−0.09, 0.36)%; p=0.23] and FPG [0.02 (−0.36, 0.40) mmol/L; p=0.91] to controls (eTable 1).

Adjusted GAM demonstrated a linear association between skin induration and FPG (effective degrees of freedom (EDF) =1.01; p < 0.001) (Fig. 2A) and a non-linear one (i.e., a non-parametric effect) between skin induration and HbA1c (EDF=1.76; p=0.003) (Fig. 2C). Of note, there was no association between IFN-γ with either FPG (EDF=1.0; p=0.58) (Fig. 2B) or HbA1c (EDF=0.94; p=0.71) (Fig. 2D). These observations were replicated in unadjusted models (Fig. E2).

### Prevalent diabetes mellitus and measures of Mtb infection

**Fig. 3:**
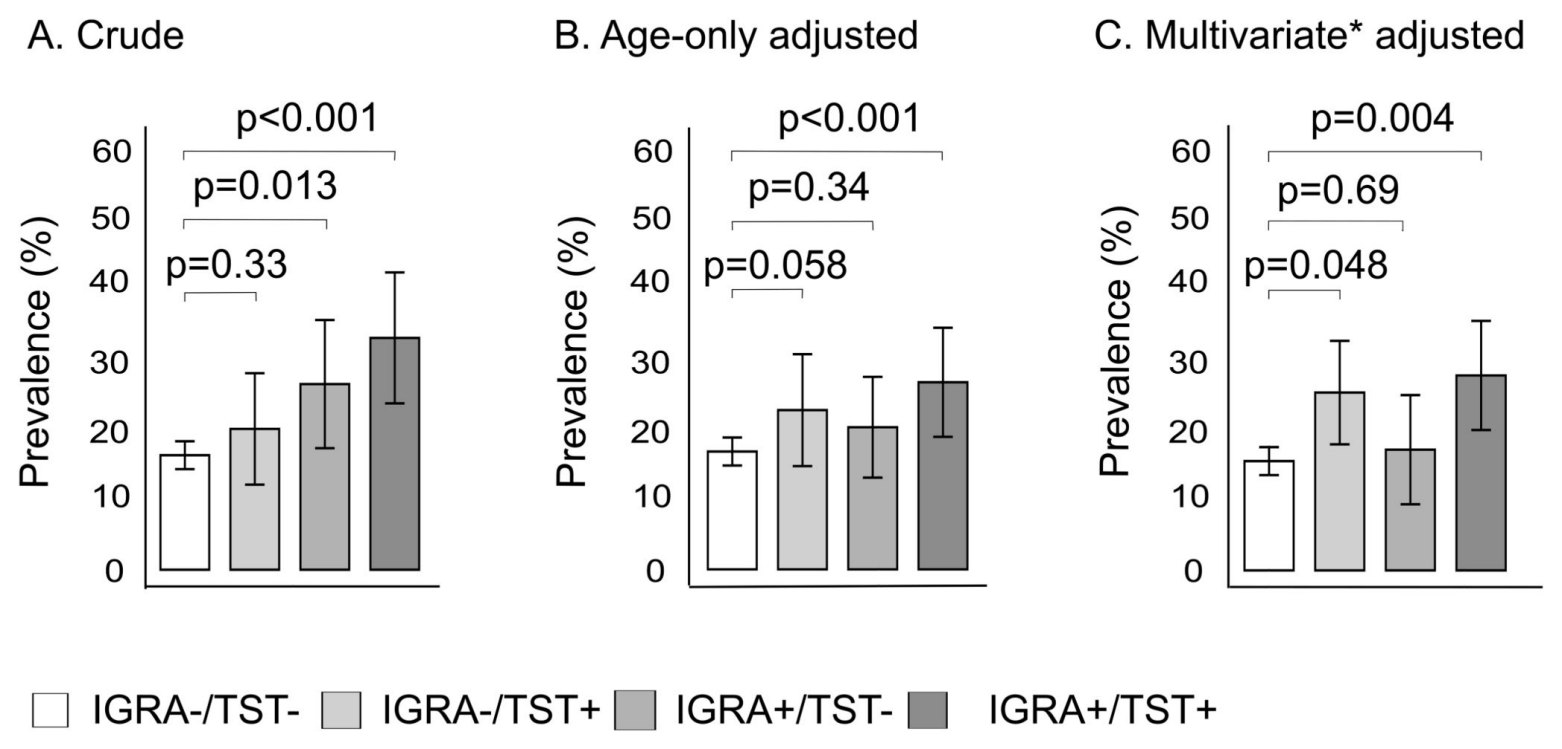
(A) Crude, (B) age- and (C) multivariate-adjusted prevalence of diabetes mellitus stratified by mycobacterial sensitization status, unweighted US NHANES 2011–2012 sample. * Prevalence estimated from GAM adjusted for age, sex, race, health insurance coverage, household poverty income ratio (PIR), waist circumference and smoking status. Mycobacterial sensitization is defined by tuberculin skin testing (TST) and interferon-γ release assay (IGRA).

The crude prevalence (95%CI) of diabetes was higher in participants with LTBI who were either IGRA+/TST+ (32.6 [23.4, 41.8]%; p < 0.001) or IGRA+/TST- (26.1 [19.1, 35.1]%; p=0.013) than among controls without LTB (16.1 [14.1, 18.1]%) (Fig. 3A). Following partial (Fig. 3B) and full (Fig. 3C) covariate adjustment, only in LTBI defined by skin induration with (IGRA+/TST+) or without IFN-γ reactivity (IGRA-/TST+) was diabetes prevalence significantly higher compared to controls without TB infection. For example, diabetes prevalence in multivariate adjusted models was 27.3 [19.7, 34.9]% (p=0.004) for participants with IGRA+/TST+ and 24.9 [17.6, 32.2]% (p=0.048) for those with IGRA-/TST+, versus 15.3 [13.3, 17.3]% for participants without TB (IGRA-/TST-) (Fig. 3C).

These results were replicated in GLM analyses (Fig. E3). Participants with LTBI defined by positive IGRA without skin induration (IGRA+/TST-) had a similar diabetes prevalence to TB-uninfected controls (adjusted prevalence ratio (adjPR): 1.0 [0.64, 1.56]; p=0.32). Those with LTBI defined by skin induration, with (adjPR: 1.41 [1.03, 1.91]) or without (adjPR: 1.44 [1.01, 2.13]) positive IGRA, had nearly 1.5 times higher diabetes prevalence than controls (IGRA-/TST-).

**Fig. 4:**
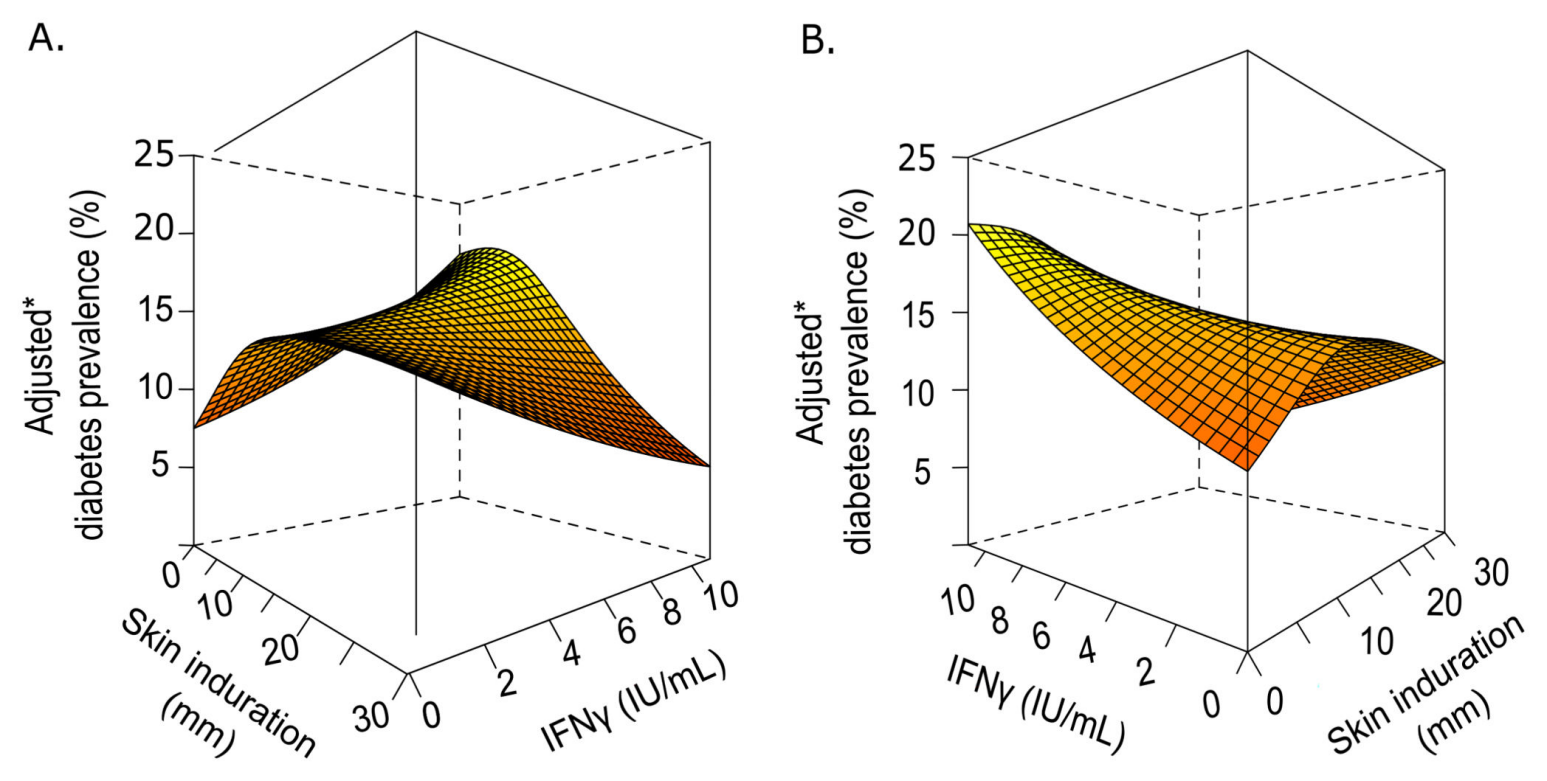
Three-dimensional perspective plot of adjusted diabetes mellitus prevalence, skin induration and IFN-γ, unweighted US NHANES 2011–2012 sample. * Prevalence estimated from general additive models adjusted for age, sex, race, health insurance coverage, household poverty income ratio (PIR), waist circumference and smoking status.

The joint effects of skin induration and IFN-γ reactivity on diabetes prevalence are presented as a 3D perspective plot in Fig. 4. At any given level of IFN-γ, the relationship between adjusted diabetes prevalence and TST induration was generally an inverted “U” shape. Diabetes prevalence peaked at a TST induration of approximately 10 mm. In contrast, diabetes prevalence was largely constant across IFN-γ levels for any given level of skin induration.

### Sociodemographic and other determinants of diabetes mellitus prevalence

The other significant determinants of prevalent diabetes were race/ethnicity, household poverty and central obesity (Fig. E3). Compared to those of Hispanic race/ethnicity, non-Hispanic whites, for example, had less prevalent diabetes (adjPR: 0.64 [0.48, 0.84]. Each 5 cm increase in waist circumference was associated with a 3% relatively higher diabetes prevalence (adjPR 1.03 [1.03, 1.04]), while living in poverty was associated with a nearly 30% relative increase (adjPR 1.28 [1.04, 1.57]) in diabetes prevalence.

### Secondary analysis

In participants with LTBI, skin induration was positively, albeit weakly, associated with HbA1c (Kendall’s Tau: 0.1; p < 0.05) (Fig. E4) in analyses exploring how glucose indices might impact measures of *Mtb* sensitization. This association, however, disappeared following confounder adjustment in generalized additive modeling (Fig. E4). We also found no significant relationship between IFN-γ and worsening hyperglycemia (assessed by either FPG or HbA1c), both with and without confounder adjustment.

## Discussion

In this cross-sectional study among U.S adults, we investigated relationships between glucose metabolism indices and measures of *Mtb* exposure. Specifically, we contrasted the associations between each of (i) skin induration in TST and (ii) IFN-γ reactivity from IGRA, with FPG, HbA1c and prevalent diabetes. We found that the association between LTBI and measures of glycemia, including diabetes, was pronounced when *Mtb* sensitization was assessed by skin induration and not by IFN-γ reactivity. These results were consistent across the different modeling approaches. We speculate that the inconsistent associations between these two assays and dysglycemia may point to a mechanistic role for innate rather than acquired immunity. Regardless, these results highlight the need for detailed immunophenotyping to more fully understand the mechanisms leading from TB to diabetes. In the light of these findings, population and clinical studies examining LTBI and especially its cardiometabolic consequences may have to consider how LTBI is evaluated in the absence of a gold standard.

Infection with *Mtb* evokes dynamic innate and adaptive cellular and humoral immune responses, and currently available tests capture this complexity to a very limited extent.^1,16,17^ Skin induration is an in vivo delayed-type hypersensitivity reaction^18^ while IGRA measures *ex vivo* IFN-γ reactivity to ESAT-6, CFP-10 and TB7.7(p4) by immune cells.^17^ Thus, TST assesses a much wider range of both innate and acquired immune components^18^ than IGRA, which represents a narrow, antigen-specific T-cell response. On the other hand, it is immune dysregulation in TB that is postulated to drive insulin resistance, hyperglycemia and eventual diabetes.^4,6^ The TST, and not IGRA, is arguably, therefore, more likely to reflect some of the immune effectors that are involved in and/or are correlated with glucose dysregulation in TB. Alternatively, IFN-γ may not be involved in TB’s potential diabetogenecity. This may be notwithstanding the fact that IFN-γ is almost invariably detected as protein or mRNA at sites of human *Mtb* infection and in *ex vivo* responses of leucocytes to mycobacterial antigens.^1,19^ This would be consistent with our finding of lack of associations between IFN-γ and any glucose biomarker.

The insufficiency of TST and IGRA to detect replicating infection is well recognized as T-cell memory can persist following *Mtb* clearance.^1^ Nonetheless, the discordance of TST and IGRA among the *Mtb* sensitized may also represent underlying variations in immunophenotype, ^16,20^ which in turn, may translate into different biological pathways and vulnerabilities to clinical diseases and their outcomes. Auld *et al*., (2013), for example, showed that a negative TST in active pulmonary TB was associated with increased mortality risk.^21^ More recently, distinct patterns and frequencies of regulatory CD4+ T-cells (Treg) were demonstrated among individuals with discordant IGRA and TST reactivity.^20^ Treg cells are involved in self-tolerance, auto-immunity and modulation of cell-mediated immunity in the presence of pathogenic bacteria, among other functions.^22^ Treg cells are also implicated in the development of diabetes and its progression.^23^ Whether these putative relationships between discordant IGRA and TST reactivity, their immunobiology correlates, including Treg cells, and diabetes risk are reflected in our present results will require further study.

Diabetes can suppress cell-mediated immune responses.^24^ Compared to TB alone, TB with comorbid diabetes has been associated with impaired differentiation and function of Th1 cells, including their production of IFN-γ.^25^ Poor agreement between TST and IGRA has been shown in diabetics.^26^ In a study of Tanzanian TB patients and non-tuberculosis controls, diabetes was associated with reduced IFN-γ release.^27^ The authors concluded that the validity of IFN-γ tests for LTBI may be questionable in individuals with diabetes. This might explain, in part at least, our finding of no association between IGRA+ and diabetes. Mitigating against this, however, is that we would also expect an inverse relationship between (continuous) IFN-γ and either FPG or HbA1c. Our secondary analysis did not however find worsening dysglycemia to be accompanied by declining IFN-γ reactivity.

Regardless, our findings may shed some light on contradictory findings of associations between LTBI and cardiometabolic diseases.^28,29,30^ Hitherto, discrepant findings have been attributed to heterogeneity of study settings, populations and methods.^30^ Discordance of TST and IGRA has not been considered. We here demonstrate for the first time differential associations between measures of *Mtb* sensitization with diabetes. In turn, this also brings to the fore the need to transcend the traditional dichotomy of active or symptomatic and latent or asymptomatic TB and recognize *Mtb* infection as an immunological, pathological and clinical spectrum.^1,2^ A recent proposal, for example, re-classifies asymptomatic TB as either (i) *Mtb infection*, defined by viable mycobacteria and an associated host response, or (ii) *non-infectious subclinical tuberculosis*, when there is also macroscopic pathology.^2^ However, our lack of operational state-specific diagnostics means that no tuberculosis-as-spectrum model is presently useful clinically or programmatically.

### Strengths and limitations

Our study is among the first to explore how discordant IGRA and TST relate to glucose metabolism measures. Our results have wide generalizability as the study was population-based with a sample drawn to reflect the diversity of the US population. Facility-based studies and/or with narrowly selected participants are more common in the field. Data on TB-related symptoms, chest radiographs and sputum examinations in conjunction with TST and IGRA would have enabled better stratification of the *Mtb* sensitized into those who have eliminated TB infection, controlled TB infection and subclinical TB infection.

As a cross-sectional study, threats from residual confounding and potential misclassification biases warrant caution with inferences. Similarly, we lacked information on other important covariates like comorbidities and BCG vaccination data. Whereas IGRA is specific for *Mtb*, low TST reactivity would not distinguish *Mtb* sensitization from exposure to non-tuberculosis mycobacterium (NTM), including *Mycobacterium bovis* in BCG vaccines. This cross-reactivity raises likelihood of TST false-positivity in our analysis. This is relevant because NTM have also been linked with diabetes.^31^ Neither did we have data to robustly distinguish type 1 from type 2 diabetes. However, the consistency of our primary and sensitivity analyses does give some reassurance.

## Conclusion

The association between LTBI and measures of glycemia, including diabetes, is pronounced when *Mtb* sensitization is assessed by skin induration and less by IFN-γ release. This suggests the involvement of innate immune mediators as opposed to acquired T-cell responses, as determined by the *ex vivo* IFN-γ release assay. Whereas detailed immune mechanistic studies will be required to more fully understand the mechanistic pathways from TB to dysglycemia, and diabetes, population and clinical studies examining LTBI and its consequences, especially cardiometabolic sequalae, may have to consider its diagnostic approaches.

## Supplementary Material

Supplementary data associated with this article can be found in the online version at doi:10.1016/j.jinf.2025.106496.

Supplementary appendix

## Figures and Tables

**Table 1:** Characteristics of participants stratified by mycobacterial sensitization status, unweighted US NHANES 2011–2012 sample.

Characteristic	Mycobacterial sensitization status^a^						
	IGRA-/TST-	IGRA-/TST+	P value^†^	IGRA+/TST-	P value^‡^	IGRA+/TST+	P value^§^
Number	1787	101		92		99	
Skin induration (mm)							
Median (IQR))	0.0 (0.0, 0.0)	13.0 (11.3, 14.7)	< 0.001	0.0 (0.0, 6.8)	0.98	15.3 (12.0, 17.5)	< 0.001
Mean (SD)	0.67 (1.9)	13.5 (3.0)	< 0.001	2.7 (3.8)	< 0.001	15.5 (3.6)	< 0.001
IFN*_γ_* (IU/mL)							
*TB antigen tube*							
Median (IQR)	0.05 (0.03, 0.09)	0.11 (0.07, 0.2)	0.17	1.3 (0.6, 3.1)	< 0.001	3.9 (1.0, 11.0)	< 0.001
Mean (SD)	0.08 (0.11)	0.15 (0.16)	0.59	2.7 (3.2)	< 0.001	5.1 (4.4)	< 0.001
*Nil tube*							
Median (IQR))	0.04 (0.03, 0.07)	0.06 (0.04, 0.10)	< 0.001	0.06 (0.04, 0.11)	< 0.001	0.06 (0.04, 0.10)	< 0.001
Mean (SD)	0.06 (0.10)	0.10 (0.14)	0.008	0.12 (0.21)	< 0.001	0.12 (0.29)	< 0.001
*Sociodemographic*							
Age (years)	47.8 (17.7)	47.7 (15.1)	0.94	57.1 (16.7)	< 0.001	56.3 (14.6)	< 0.001
Female sex	924 (51.7%)	49 (48.5%)	0.53	32 (34.8%)	0.002	43 (43.4%)	0.11
Race/ethnicity							
Hispanic	320 (17.9%)	39 (38.6%)	< 0.001	31 (33.7%)	< 0.001	46 (46.5%)	< 0.001
Non-Hispanic white	788 (44.1%)	10 (9.9%)		21 (22.8%)		4 (4.0%)	
Non-Hispanic black	439 (24.6%)	22 (21.8%)		17 (18.5%)		22 (22.2%)	
Non-Hispanic Asian/other	240 (13.4%)	30 (29.7%)		23 (25.0%)		27 (27.3%)	
No health insurance	404 (22.6%)	34 (33.7%)	0.011	25 (27.2%)	0.31	28 (28.3%)	0.19
Median household PIR	2.0 (1.0, 3.9)	1.7 (0.9, 3.1)	0.52	1.8 (0.9, 3.6)	0.61	1.7 (0.9, 3.1)	0.40
Household PIR < 1.3	607 (36.5%)	33 (38.4%)	0.94	33 (40.2%)	0.55	36 (40.4%)	0.49
*Cardiometabolic*							
Hypertension	736 (41.2%)	38 (37.6%)	0.48	44 (47.8%)	0.21	50 (50.5%)	0.069
Systolic BP (mmHg)	122.5 (17.7)	121.9 (15.7)	0.76	123.6 (18.1)	0.57	127.5 (20.0)	0.007
Diastolic BP (mmHg)	70.5 (11.3)	71.7 (10.4)	0.33	67.5 (12.7)	0.015	71.5 (10.4)	0.43
Current medication use							
Aspirin	20 (1.1%)	0 (0.0%)	-	1 (1.1%)	0.98	2 (2.0%)	0.43
Oral anticoagulants	35 (2.0%)	0 (0.0%)	-	2 (2.2%)	0.85	0 (0.0%)	-
Insulin/oral hypoglycemics	149 (8.3%)	11 (10.9%)	0.38	16 (17.4%)	0.004	16 (16.2%)	0.009
Statins	332 (18.6%)	16 (15.8%)	0.49	29 (31.5%)	0.003	27 (27.3%)	0.034
Current smoker	359 (20.1%)	21 (20.8%)	0.89	16 (17.4%)	0.53	22 (22.2%)	0.61
BMI (kg/m^2^)	29.0 (6.7)	28.4 (6.5)	0.39	28.3 (8.0)	0.34	29.4 (6.5)	0.53
Waist circumference (cm)	98.8 (16.3)	95.8 (14.6)	0.050	98.3 (17.7)	0.78	101.1 (15.7)	0.19

Values are number (%), mean (SD) or median (IQR).

† P values are for comparisons between TB uninfected controls (IGRA-/TST-) versus (i) IGRA-/TST+

‡ (ii) IGRA+/TST-

§ (iii) IGRA+/TST+

P values are Bonferroni-adjusted with statistical significance level (α’) < 0.0167.

PIR = poverty-income ratio; BP = blood pressure; BMI = body mass index.

a Mycobacterial sensitization defined by tuberculin skin testing (TST) and interferon-γ release assay (IGRA).

## Data Availability

Data are publicly available at https://www.cdc.gov/nchs/nhanes/index.htm. Programming code is available upon request.
