# Supplementary material for: Discordance between measures of Mycobacterium tuberculosis sensitization and type 2 diabetes mellitus in the United States (NHANES): A population-based cohort study
Source: J Infect. Author manuscript; Available in PMC 2025 Nov 18. (PMC7618374; doi:10.1016/j.jinf.2025.106496)
Supplement: Supplementary appendix [file EMS210686-supplement-Supplementary_appendix.docx]

**Supplementary appendix**

**Discordance between measures of Mycobacterium tuberculosis sensitization and type 2 diabetes mellitus in the United States (NHANES): a population-based cohort study**

**Table of contents Page**

Table E1 1

Table E2 2

Figure E1 3

Figure E2 4

Figure E3 6

Figure E4 7

**Table E1. Adjusted associations between fasting plasma glucose and HbA1c and mycobacterial sensitization status and other participant characteristics, unweighted US NHANES 2011-2012 sample.**

| **Characteristic** | **Adjusted^α^ mean difference** | | | |
| --- | --- | --- | --- | --- |
|  | **Fasting plasma glucose (mmol/L)** | | **HbA1c (%)** | |
|  | **Estimate (95%CI)** | **P value** | **Estimate (95%CI)** | **P value** |
| *M.tb* sensitization status |  |  |  |  |
| IGRA-/TST- | Reference |  | Reference |  |
| IGRA-/TST+ | 0.48 (0.12, 0.84) | 0.008 | 0.25 (0.05, 0.47) | 0.017 |
| IGRA+/TST- | 0.02 (-0.36, 0.40) | 0.91 | 0.14 (-0.09, 0.36) | 0.23 |
| IGRA+/TST+ | 0.22 (0.0, 0.43) | 0.051 | 0.26 (0.04, 0.43) | 0.027 |
| 5-year age change | 0.11 (0.08, 0.13) | <0.001 | 0.02 (0.01, 0.02) | <0.001 |
| Sex |  |  |  |  |
| Female | Reference |  | Reference |  |
| Male | 0.15 (-0.01, 0.32) | 0.063 | -0.01 (-0.10, 0.08) | 0.79 |
| Race/ethnicity |  |  |  |  |
| Hispanic | Reference |  | Reference |  |
| Non-Hispanic white | -0.29 (-0.51, -0.07) | 0.009 | -0.27 (-0.39, -0.14) | <0.001 |
| Non-Hispanic black | -0.06 (-0.30, 0.17) | 0.61 | 0.03 (-0.11, 0.17) | 0.69 |
| Non-Hispanic Asian/other | 0.16 (-0.10, 0.43) | 0.23 | 0.08 (-0.08, 0.23) | 0.34 |
| Has health insurance | Reference |  | Reference |  |
| No health insurance | -0.14 (-0.34, 0.05) | 0.15 | -0.05 (-0.16, 0.07) | 0.43 |
| Median household PIR |  |  |  |  |
| Household PIR ≥1.3 | Reference |  | Reference |  |
| Household PIR <1.3 | 0.24 (0.08, 0.41) | 0.004 | 0.14 (0.04, 0.23) | 0.007 |
| 5cm waist circumference change | 0.03 (0.02, 0.03) | 0.000 | 0.02 (0.01, 0.03) | <0.001 |
| Smoking status |  |  |  |  |
| Non-smoker | Reference |  | Reference |  |
| Smoker | 0.07 (-0.13, 0.27) | 0.49 | 0.09 (-0.2, 0.21) | 0.11 |

Values are β-coefficients (95% CI) from linear regression models adjusted for age, sex, race, health insurance coverage, household poverty income ratio (PIR), waist circumference and smoking status.

PIR = poverty-income ratio.

**Table E2. Unadjusted associations between fasting plasma glucose and HbA1c and measures of mycobacterial sensitization, unweighted US NHANES 2011-2012 sample.**

| **Smooth terms** | **Fasting plasma glucose** | | **HbA1c** | |
| --- | --- | --- | --- | --- |
|  | **EDF** | **P-value** | **EDF** | **P-value** |
| s(IFN-γ) | 1.0 | 0.13 | 1.0 | 0.17 |
| s(TST) | 1.0 | 0.016 | 1.0 | 0.009 |
| s(IFN-γ, TST) | 1.0 | 0.17 | 1.58 | 0.079 |

Generalized additive models of FPG and HbA1c on TST and IFN-γ, and including TST/IFN-γ interaction with no confounder adjustment:

*Biomarker =s_1_.(IFN-γ) + s_2_.(TST) + s_3_.(IFN-γ, TST)*

EDF = effective degrees of freedom.

**Figure E1. Flow chart of participants included in final analytic sample using US NHANES 2011-2012.**

Participants in 2011-2012 NHANES

N = 9,756

Excluded from analysis (N = 2,155):

Missing FPG (n=2,132)

Missing HbA1c (n=11 )

Missing/invalid TST results (n=1,300)

Missing/invalid IGRA results (n=579)

Age <20 years or missing (n=3,088)

Complete analytic dataset

N = 2,079

Valid IGRA and TST results

N = 4,234

Adults ≥20 years old

N = 5,561

**Figure E2. Predicted associations between measures of mycobacterial sensitization and fasting plasma glucose and HbA1c among participants with latent tuberculosis infection, unweighted US NHANES 2011-2012 sample.**

IGRA = interferon-γ release assay; TST = tuberculin skin testing; FPG = fasting plasma glucose.

IGRA-/TST-

N = 1,787

IGRA-/TST+

N = 101

IGRA+/TST+

N = 99

IGRA+/TST-

N = 92

* EDF = effective degrees of freedom.

Generalized additive models (GAM) of TST and IFN-γ on FPG and HbA1c , and including FPG/HbA1c interaction. Analyses limited to participants with either IGRA+ and/or TST+.

*TST (or IFN-γ) =s_1_.(FPG) + s_2_.(HbA1c) + s_3_.(FPG, HbA1c) + β_1_.age + β_2_.sex + β_3_.race + … β_4_.smoking*

Gray areas represent 95% CI. Adjusted for age, sex, race, health insurance coverage, household poverty income ratio (PIR), waist circumference and smoking status.

**
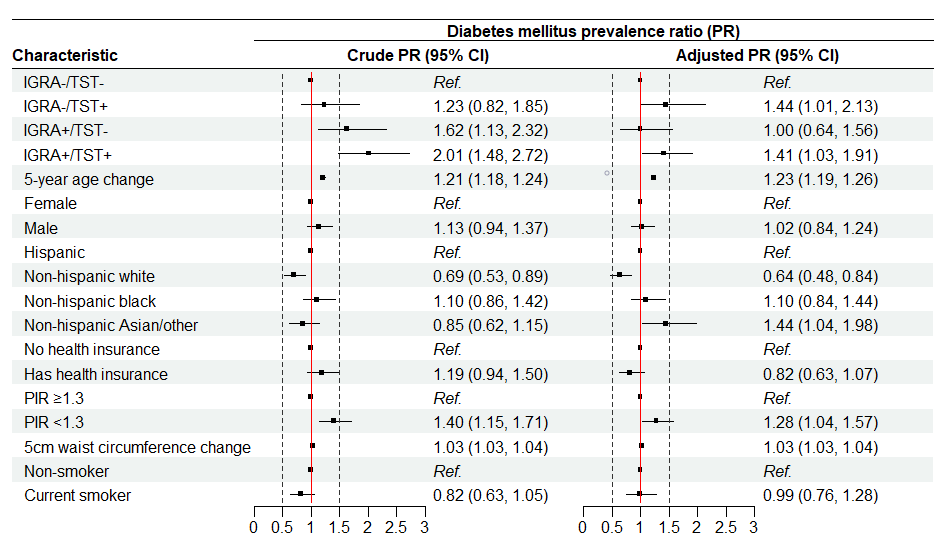
Figure E3. Crude and adjusted prevalence ratio of diabetes mellitus according to mycobacterial sensitization status and other participant characteristics, unweighted US NHANES 2011-2012 sample.**

Prevalence ratios (PR) were estimated from univariate (*crude*) and multivariable (*adjusted**) log binomial regression models.

* Adjusted for age, sex, race, health insurance coverage, household poverty income ratio (PIR), waist circumference and smoking status.

**Figure E4. Correlations between fasting plasma glucose and HbA1c and measures of mycobacterial sensitization among participants with latent tuberculosis infection, unweighted US NHANES 2011-2012 sample.**

Strength of correlation determined to be either weak (0–0.39), moderate (0.40–0.69), or strong (0.70–1.0).

** P-value significant at <0.05. Analyses limited to participants with either IGRA+ and/or TST+.
